# Deep machine learning provides state-of-the-art performance in image-based plant phenotyping

**DOI:** 10.1093/gigascience/gix083

**Published:** 2017-08-23

**Authors:** Michael P. Pound, Jonathan A. Atkinson, Alexandra J. Townsend, Michael H. Wilson, Marcus Griffiths, Aaron S. Jackson, Adrian Bulat, Georgios Tzimiropoulos, Darren M. Wells, Erik H. Murchie, Tony P. Pridmore, Andrew P. French

**Affiliations:** 1 School of Computer Science, University of Nottingham, Jubilee Campus, Wollaton Road, Nottingham, NG8 1BB, UK; 2 School of Biosciences, University of Nottingham, Sutton Bonington Campus, Nr Loughborough, LE12 5RD, UK; 3 Centre for Plant Sciences, Faculty of Biological Sciences, University of Leeds, Leeds, LS2 9JT, UK

**Keywords:** Phenotyping, deep learning, root, shoot, QTL, image analysis

## Abstract

In plant phenotyping, it has become important to be able to measure many features on large image sets in order to aid genetic discovery. The size of the datasets, now often captured robotically, often precludes manual inspection, hence the motivation for finding a fully automated approach. Deep learning is an emerging field that promises unparalleled results on many data analysis problems. Building on artificial neural networks, deep approaches have many more hidden layers in the network, and hence have greater discriminative and predictive power. We demonstrate the use of such approaches as part of a plant phenotyping pipeline. We show the success offered by such techniques when applied to the challenging problem of image-based plant phenotyping and demonstrate state-of-the-art results (>97% accuracy) for root and shoot feature identification and localization. We use fully automated trait identification using deep learning to identify quantitative trait loci in root architecture datasets. The majority (12 out of 14) of manually identified quantitative trait loci were also discovered using our automated approach based on deep learning detection to locate plant features. We have shown deep learning–based phenotyping to have very good detection and localization accuracy in validation and testing image sets. We have shown that such features can be used to derive meaningful biological traits, which in turn can be used in quantitative trait loci discovery pipelines. This process can be completely automated. We predict a paradigm shift in image-based phenotyping bought about by such deep learning approaches, given sufficient training sets.

## Background

The large increase in available genomic information in plant biology has led to a need for truly high-throughput phenotyping workflows to bridge the increasing genotype-phenotype gap. Image analysis has become a key component in these workflows [1], where automated measurement and counting have allowed for increased throughput and unbiased, consistent measurement systems. Machine learning has proven to be one of the most flexible and powerful analysis techniques, with approaches such as Support Vector Machines [2] and Random Forests [3] achieving the highest success rates to date. Whilst these techniques provide considerable success in many situations [4], their performance is saturating and often falls short of the high accuracy required for fully automated systems. However, with careful crafting of features, these approaches can have practical application still. What deep learning promises is the learning of the features themselves; often, given sufficient training data, allowing for increases of accuracy.

Before introducing deep learning, it is helpful to first consider traditional machine learning techniques applied to bioimage analysis. It is generally assumed that raw images will contain too much information for a machine learning approach to efficiently process. For this reason, much of the established research in this field involves pre-computation of domain-specific image *features*; hand-crafted, for example, to detect areas of high contrast such as types of edges and corners. This pre-processing is intended to capture enough information to represent classes of objects but contain significantly fewer dimensions than the full set of original image pixels [4]. The output of this feature detection is passed into a classifier, where classes (here, phenotypic traits) can be efficiently separated. Crucially, the choice of features is left to the designer and is often limited to existing sets, popular in the literature. These hand-crafted features are not guaranteed to provide the subsequent learning algorithm with the optimal description of the data, which in turn will reduce its effectiveness. It is easy to accidentally limit the application of the algorithm to specific tasks; an approach that performs well in one task may fail to perform in a different task. There is, therefore, a motivation to produce more general learning approaches.

**Figure 1:**
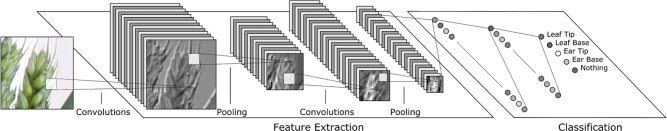
A simplified example of a CNN architecture operating on a fixed size image of part of an ear of wheat. The network performs alternating *convolution* and *pooling* operations (see the online methods for details). Each convolutional layer automatically extracts useful features, such as edges or corners, outputting a number of feature maps. Pooling operations shrink the size of the feature maps to improve efficiency. The number of feature maps is increased deeper into the network to improve classification accuracy. Finally, standard neural network layers comprise the classification layers, which output probabilities for each class.

Early general approaches include the biologically inspired artificial neural networks (ANNs), which use a set of simulated neuron-like connections and transfer inputs via a set of learnt functions to a series of outputs. These represent a set of activations propagating through a network structure, triggered by input data, and resulting in an output activation pattern. ANNs typically use three layers, one for input, a hidden internal layer, and an output layer. Modern deep learning approaches extend this concept and may contain many additional layers of artificial neurons (hence the term *deep*) and with increased complexity bring significantly increased discriminative power [5]. Cutting edge algorithms and computational hardware have bought the training time for such networks down to practical levels achievable in most labs. Convolutional neural networks (CNNs) specialize this representation further, replacing the neuron layers with feature-detecting convolution layers (biologically inspired by the organization of the visual field) [6], before finishing with traditional ANN layers to perform classification (Fig. 1). CNNs have been quickly adopted by the computer vision community, but have also recently been used successfully in the life sciences [7] and medicine [8].

The CNN transforms feature maps from previous layers, creating a rich hierarchy of features that can be used for classification. For example, while the initial layer may compute simple primitives such as edges and corners, deeper into the network feature maps, based on these will highlight groups of corners and edges. Deeper still, feature maps may contain complex arrangements of features representing real-world objects [9]. It is important to note that these features are learnt by the CNN training algorithms and are not hand-coded.

Modern CNNs will typically use many layers, which makes training the networks complex, often requiring hundreds, sometimes thousands, of images to train to the desired accuracy [10]. However, once trained, their accuracy is unrivaled, and they can be transferred to other related domains by re-training using significantly fewer images [11]. A CNN is trained by iteratively passing example images containing the objects to be detected into the network and adjusting the network parameters based on the results. The values of the convolutional filters are automatically adjusted to improve the result the next time a similar image is seen, a process that is repeated for as many images as possible.

**Figure 2:**
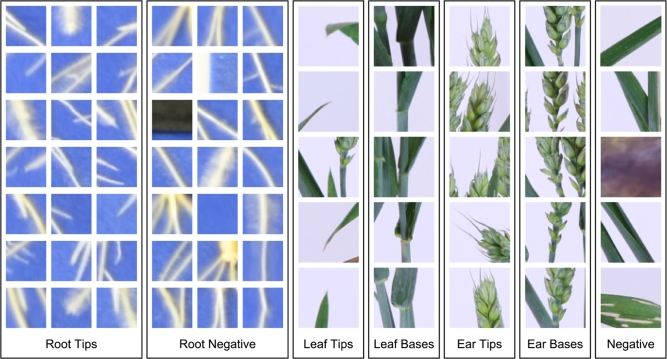
Example training and validation images from our root tip and shoot feature datasets. Positive samples were taken at locations annotated by a user. Negative samples were generated on the root system and at random for the root images, and on computed feature points on the shoot images.

To demonstrate the effectiveness of this deep learning approach, we first trained 2 separate CNNs on 2 tasks central to plant phenotyping, framed as classification problems. In the first, we address the following question: given a small section of a root system image, can a CNN identify if a root tip is present? The architecture of a root system is an important aspect of its physiological function; the root system's structure allows it to access different nutrients and water within the soil profile. In phenotyping, particularly with high-throughput 2D approaches, identifying features such as root tips represents the rate-limiting step in data quantification. We prepared training image data in which some images contained root tips and some did not. This was derived from a dataset containing 2500 annotated images of whole root systems, and automatically generated classification images, by cropping at the annotated tip locations (See Fig. 2, left side). This dataset is publically available in Pound et al. [12].

In the second classification problem, given an image of a section of plant shoot, we ask: can a CNN identify biologically relevant features such as leaf and ear tips, bases, etc.? This would allow high-throughput phenotyping on an extremely large number of lines based on single images. It also allows 3D shoot structure to be linked with physiological functioning: e.g., the separation into individual leaves and organs allows us to place biologically distinct plant parts within a useful functional context (different leaves, reproductive organs). To do this, we hand-annotated 1664 images of wheat plants, labelling leaf tips, leaf bases, ear tips, and ear bases. Classification images were then automatically extracted from these images as before (see Fig. 2, right side). This dataset is also publically available in Pound et al. [12].

We then quantify the accuracy of finding the features in the 2 image sets. We also show how it is possible to localize the features within the image—answering questions such as, where are the root tips located? The Methods section explains in detail the process of preparing the networks and data and the training of the CNNs, as well as the localization approach used.

A common goal of phenotyping studies is the use of mapping populations to investigate the genetic architecture of complex traits by identifying quantitative trait loci (QTL; regions of DNA that correlate with phenotypic variations). QTL analysis is based on detecting an association between phenotype and genotypic markers; the markers are used to partition a population into genotypic groups, whereupon trait differences between the groups can be identified [13]. The collective effect of numerous genes controls the genetic variation in a quantitative trait. Identifying such QTL is of agronomic importance and feeds into the development of crop species. QTL discovery itself relies on the statistical analysis of phenotypic traits and has been limited by the lack of unbiased, high-throughput techniques to extract trait values from image sets.

Finally, then, we demonstrate that it is possible to automatically derive traits from images using these features, which can be used to identify the underlying genetic architecture by identifying QTL, a key goal of many phenotyping studies. The output of the root CNN (the detected root tips) is then used to derive simple descriptive traits automatically, which are then used in a QTL discovery process and compared to QTL discovery via a more manual approach.

### Data description

Two datasets have been used in this paper, each presenting a unique challenge to deep learning. By presenting both, we wish to highlight the wide applicability of the approach.

#### Root analysis

Bread wheat (*Triticum aestivum L*.) seeds were sieved to uniform size, sterilized, and pre-germinated before transfer to growth pouches in a controlled environment chamber (12-hour photoperiod: 20°C day, 15°C night, with a light intensity of 400 μmol m–2 s–1 PAR), as per Atkinson et al. [14]. After 9 days (two-leaf stage), individual pouches were transferred to a copy stand for imaging using a Nikon D5100 DSLR camera controlled using NKRemote software (Breeze Systems Ltd, Camberley, UK). Root system architectural traits were extracted from images of 2697 seedlings using the *RootNav* software (RootNav, RRID:SCR_015584) [15] and used to produce the input images for CNN training.

#### Shoot analysis

Wheat varieties were grown as detailed previously [16]. Plants in pots were imaged according to the protocol of Pound et al. [17]. The developmental stages of the plants in both years of trial were the same. At anthesis, wheat plants (roots and shoots) were removed from the field and taken to a photography studio located close by to prevent wilting and damage to the shoots. They were imaged using 3 fixed Canon 650D cameras, with a minimum of 40 images per plant. Images were captured using a revolving turntable, including a fixed size calibration target. This target is used to facilitate 3D reconstruction, which does not feature in this work.

Further details on preparation of the image data for the networks can be found in the Methods section.

### Analyses

#### Classification

Once networks are built and training has been completed (see the Methods section), the learned parameters of the network are then stored and can be used to perform classification when required. The final accuracy of the networks described in this paper is the result of a final evaluation over all validation images once training was stopped. Our CNN models, learned parameters, and all the related scripts for training and validation will be made publically available [12].

For both the root and shoot data, we randomly separated 80% of the data into a training set, and 20% remained for validation. To evaluate the accuracy of each network, we ran each validation image through the network, obtaining the likelihood of each class. These were then compared to the true label for each image to ascertain whether the network had correctly classified the image. Based on this, the accuracy of the root tip detection network was found to be 98.4%. The shoot dataset, containing 4 classes of shoot features, along with numerous instances of cluttered, non-plant background, represents an even more challenging task. In this case, the shoot network successfully classified 97.3% of images. In both cases, CNNs here have out-performed recent state-of-the-art systems (e.g., accuracies of 80–90% have been typical) [2, 18]. Accuracy results for individual classes can be seen in Table 1. Note also that both these scenarios are much more challenging than typical successes seen to date as the images involved are much less constrained.

**Table 1:** Classification results for both root and shoot datasets

Feature	Correctly classified	Misclassified	Accuracy (%)
Root tip	2904	73	97.5
Root tip negative	5687	65	98.9
**Total/average**	**8591**	**138**	**98.4**
Feature	Correctly classified	Misclassified	Accuracy (%)
Leaf tip	2225	113	95.2
Leaf base	2299	52	97.8
Ear tip	686	15	97.9
Ear base	765	23	97.1
Shoot negative	6110	136	97.8
**Total/average**	**12 085**	**339**	**97.3**

Leaf tips represent the hardest classification problem in the datasets, with large variations in orientation, size, shape, and colour. In all cases, the accuracy has remained above 95%, with the average accuracy of both networks above 97%. The root tip network performs marginally better overall, perhaps to be expected due to the simpler nature of the image data. Complete confusion matrices can be found in Additional file 3.

#### Localization

As well as *identifying* features by classifying image crops, it is necessary in quantitative phenotyping to *locate* the features within the larger image. For example, reliably identifying the locations of root tips is a bottleneck in automated root system analysis [15] and is often omitted from image analysis software due to the challenges localization presents. As another example, locating seed feature points must occur before automated tracing in RootTrace (RootTrace, RRID:SCR_015585) [19]. Localization of the different biological feature classes for a shoot is vital in capturing the architecture of the plant, essential for phenotyping. We also later show that automated localization of such features can be used to identify the underlying genetic architecture of traits.

We have extended our root and shoot classifiers to perform localization by scanning over each original image, applying the respective classifier over each image at regular pixel intervals (often referred to as a stride). Selection of the stride is straightforward and is a compromise between pixel-wise accuracy of the resulting classification map and computational efficiency. A stride of 1 will produce sub-images centred on every pixel, such that images will overlap with the majority of the previous sub-image. This means that a feature visible in one image will also be visible in a number of consecutive images around it. For both the root and shoot system images, we chose a stride of 4, which results in a single scan that takes under 2 minutes, and yet will output a classification map showing each feature location clearly. The scripts we used to perform this classification and repeat this automatically over any number of images can be downloaded alongside our models.

**Figure 3:**
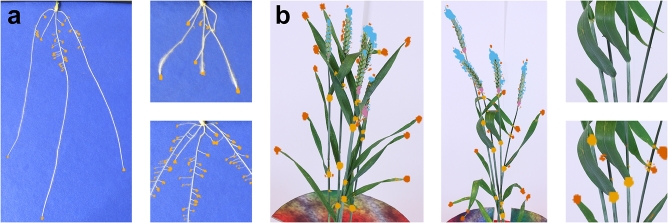
Localization examples. Images showing the response of our classifier using a sliding window over each input image. (**a**) Three examples of wheat root tip localization. Regions of high response from the classifier are shown in yellow. (**b**) Two examples of wheat shoot feature localization. Regions of high response from the classifier for leaf tips are highlighted in orange, leaf bases in yellow, ear tips in blue, and ear bases in pink. A portion of the second image has been zoomed and shown with and without features highlighted. More images can be seen in Additional file 1.

As the output of the network is a set of class probabilities, pixels observed as above a likelihood threshold are marked as belonging to a specific class (see Fig. 3).

#### Testing localization accuracy

We have tested the real-world accuracy of our localization step by measuring the proportion of location windows containing false-positives or -negatives. This testing was performed on unseen test data, comprising 20 images for roots and 20 for shoots. In both cases, no images, or parts of these images, had been used in the training or validation of either network. Accuracy was measured as the percentage of pixels that were correctly classified as either true-positives or true-negatives. False-positives were determined as those pixels that were classified as a feature but were outside of a radius around any ground truth features. This radius was set as half of the classification window size, in which any feature should be visible. False-negatives were those pixels within the same radius of a ground truth feature that were not correctly classified as those features. Separate results for roots and shoots, and for each class, can be seen in Table 2; test images and output can be seen in Additional file 2.

**Table 2:** Testing results for our image scanning approach over 20 unseen root images and 20 unseen shoot images

	Feature	False positive (%)	False negative (%)	Feature accuracy (%)
Roots	Root tip	0.03	0.12	99.85
Shoots	Leaf tip	0.24	0.12	99.64
	Leaf base	0.22	0.10	99.68
	Ear tip	0.08	0.02	99.91
	Ear base	0.11	0.05	99.85

Feature accuracy is the number of true-positive and true-negative pixels, divided by the total number of pixels over the 20 images. Actual testing images and results can be seen in Additional file 2.

The accuracy of the root tip location is 99.85% and the accuracy of the shoot feature location is 99.07% when totalled over all features. Accuracy that is higher than that of the base classifiers presented earlier (Table 1) is not surprising. During training of the networks, we generated particularly challenging negative examples of image features; these examples comprise only a very small fraction of each whole, real-world image. The scripts used for testing will be made available alongside our models [12].

### Application to QTL discovery

So far we have demonstrated the success of the approach in locating features in images. Here, we wish to show the power of a complete pipeline for phenotyping and discovery. We will use traits derived from features automatically discovered via our deep learning approach to identify significant QTL for the root system, highlighting the power of the approach for genetic discovery. As a baseline, using the semi-automated software package RootNav [15], root traits were manually determined from 1709 images of the seedling root systems of 92 members of a wheat doubled haploid mapping population [14]. These trait values were then used to identify 29 root QTL [14], representing 5 classes of trait. This same image set formed part of the training dataset for the root tip detection CNN. We will here consider only traits related to root tips as this is the feature our network specializes in, but of course different and additional features could be learned in the future.

The output of the root tip CNN after scanning over an image is a heatmap of high-likelihood tip locations. This was adapted to produce individual co-ordinates for each identified root tip. Mathematical morphology was used to erode the heat map with a 3 × 3 structuring element using 3 iterations. This removes small artefacts’ output as single pixels in the heat map and can separate some root tips that are close together. This level of erosion was chosen as a compromise between effectively removing noise and removing root tips themselves in error. A connected component algorithm was then used to find a single centroid of each foreground region, representing the most likely root tip locations. Geometrical traits were then conceived, which were derived from these recorded tip positions (listed in Table 3). Note that if detecting more than just tips of roots (perhaps the seed location or the roots themselves), much more complex and potentially informative traits could be derived. However, here we demonstrate with simple tip-based traits and use these traits to identify QTL via the same pipeline developed for the original RootNav-derived images [14]. Here we make an estimate for seed location derived from tips alone, taken as the mid-point of the top of the bounding box surrounding all seed tips. This is an estimate only, but is calculated consistently for all images.

The traits in Table 3 were then used in subsequent QTL analysis. QTL calculation and plotting of logarithm of odds (LOD) scores were conducted using R package “qtl” on best linear predictors in the first step as a single QTL model employing the extended Haley-Knott method on imputed genotypes. Significant thresholds for the QTLs were calculated from the data distribution. Final QTL LOD scores and effects were received from a multiple QTL model using the QTL detected in the initial scan. The high-density Savannah × Rialto iSelect map [20] was used, with redundant markers and those markers closer than 0.5 cM stripped out. Outputs of the analysis program R/qtl [21] are summarized in Table 4. Many of the QTL found in the original RootNav study were based on measurements of root angle and thus would not be expected to be found using parameters computable from tip positions alone; thus, these were not considered in these analyses (please see original paper for the full list) [14]. However, as can be seen in Table 3, nearly all traits related to tip location that the semi-automated RootNav approach returned were also picked up by deep learning.

**Table 3:** List of root traits derived from the tip-detection CNN output and how they were computed

Name	Description
Tip count	The sum of all connected components found
Hull area	The area of the convex hull derived from the centroids of all tips
Width/depth	The width and depth of the bounding box surrounding all tips
Width:depth ratio	Calculated as width divided by depth
Mean X/Y	The mean X and Y positions of all tips
Standard deviation X/Y	The standard deviation of the X and Y positions of all tips
Top 100/200/300px count	A count of the number of tips located in the top 100-, 200-, and 300-pixel strips below the seed position calculated above
Total length	An estimate for the length of the root system, calculated as the sum of the distances from each tip to the seed position
Centre mass X/Y	The mean X, Y position of all tips

The name is derived from the trait they can be seen to estimate or represent.

**Table 4:** QTL discovery results from user-supervised (RootNav, RN) and CNN-derived deep learning (DL) approaches

	RN				DL					
Trait	Chr	Pos	LOD	CI	Chr	Pos	LOD	CI	Additive effect	Nearest marker
Centre of mass (x)	1A	70.3	2.5	47.7–163.6						
Width/depth ratio	4D	4.8	2.7	0.8–67.6	4D	2.8	3.2	0.8–67.6	0.07	IAAV5065
Total root length	6D	4.4	24.0	2–53	6D	4.4	12.7	2–53	–2201	wsnp_Ex_c4789_8550135
Convex hull	6D	4.4	17.6	2–53	6D	4.4	17.3	2–53	–264 026	wsnp_Ex_c4789_8550135
Centre of mass (x)	6D	26	2.8	0–92.5	6D	5	17.1	2–53	–151	wsnp_Ex_c4789_8550135
Centre of mass (y)	6D	4.4	19.1	2–53	6D	4.4	10.0	0–53	–105	wsnp_Ex_c4789_8550135
Lateral count/tip count	6D	4.4	9.1	0–53	6D	4.4	10.2	0–53	–4.53	wsnp_Ex_c4789_8550135
Maximum depth	6D	4.4	22.7	2–53	6D	4.4	25.1	2–53	–388	wsnp_Ex_c4789_8550135
Maximum width	6D	4.4	6.4	0–53	6D	6	15.0	2–53	–241	wsnp_Ex_c4789_8550135
Total root length	7D	27	9.0	16–52	7D	30	3.4	16–52	–1122	Kukri_c48125_714
Lateral count/tip count	7D	29	2.4	16–101.8	7D	29	4.5	16–101.8	–2.76	wsnp_Ra_c8297_14095831
Centre of mass (x)	7D	19	2.7	16–38.8						
Convex hull	7D	34	3.5	16–62.4	7D	34	4.4	16–62.4	–123 896	Kukri_c48125_714
Maximum depth	7D	30	5.8	16–52	7D	30	6.9	16–62.4	–155	wsnp_Ra_c8297_14095831

Note there are 2 QTL identified using RN that are missed by the DL approach; all others were identified by both methods. Chr: chromosome; CI: confidence interval start and end positions; DL: deep learning; Pos: position; RN: RootNav.

Traits derived from the CNN resulted in the detection of 12 QTL; all of these coincide with loci discovered using the manual RootNav approach. The QTL on chromosome 1A for one trait, “Centre of mass (x),” was not detected using the deep learning approach but *was* found using trait values from RootNav. This trait represents the centre of mass of the root system in the horizontal direction and only varies by 11 mm across the mapping population in the RootNav data. By here estimating the seed position, this small amount of variation is not captured using the root tip positions alone, and thus the QTL is not detected. Additionally, the trait itself is likely to be of little biological relevance although it is significant in the RootNav analysis, so we include it here for completeness. Finally, it is worth noting though that a second QTL for the same trait was detected on chromosome 6D using both systems.

Extraction of phenotypic information using RootNav requires a skilled user and a considerable investment of time (the most experienced users take on average 2 minutes to process an image). The CNN-derived tip detection pipeline runs *completely* unattended, is free from operator bias, and successfully found 86% of the tip-related QTL previously identified using trait values extracted via the semi-automated RootNav pipeline. This highlights the potential for deep learning in delivering the automated, high-throughput extraction of useful data from images required for phenotyping studies.

Of course, the benefits of deep learning are only possible given sufficient quantities of representative training data. The deeper the network, the more data are required. Quality of training data and the training protocol can affect final results. Traditional machine learning may work with smaller quantities of training data due to fewer parameters having to be learnt in the models. For comparison, the root architecture dataset presented in this study has also been used with a crafted feature set and Random Forest classification in a similar phenotyping pipeline; we refer the reader to Atkinson et al. [22] for more details.

## Discussion

CNNs offer unparalleled discriminative performance in classification of images and localization tasks. Here, we have demonstrated their efficacy of not only the classification, but also localization of plant root and shoot features, significantly improving upon the state of the art. To our knowledge, this is the first demonstration of deep learning applied in the localization of plant features. The success here parallels the success of deep learning in related image analysis tasks such as leaf segmentation [23]. We have also demonstrated the ability to derive meaningful traits from simple feature detection as a demonstrator, from which we successfully identify significant QTL, corroborated by manual methods. The successful application of deep learning in QTL analysis parallels the application of traditional machine learning on a similar task [22]. To improve our own methods in future work, we will explore the application of so-called fully convolutional networks, performing segmentation directly, rather than via a scanning approach. We also hope to apply feature localization to other datasets, and in particular examine the efficacy of these techniques in field images.

Deep learning is a very general technique; CNNs can be easily applied to other challenging problems and determine useful features for classification automatically during training. Microscopy, x-ray, ultrasound, magnetic resonance imaging, or other forms of medicinal and structural imaging are all targets where deep learning will yield excellent results. Areas involving challenging, unstructured images—such as those from the field—are of particular interest for future work.

Training of CNN methods of course depends on high-quality annotations on which to train. Despite skilled biological experts performing the annotation, even here we should expect some error in the annotations, over the hundreds of images and many thousands of features. Whilst we have not quantified this error on our data, it is worth keeping in mind that we must minimize such occurrences when using CNNs and that any claim to accuracy depends on “correct” annotation—what if the network is right, and the annotator wrong? These are questions that deep learning will force us to address. Annotation is also a time-consuming process, and existing datasets will perform a key role in boot strapping new techniques and applications of deep learning. This will likely drive a renewed effort in large, publicly available datasets, including high-quality annotations.

### Potential implications

We believe that the substantial increase in throughput offered by deep learning will lead to an improvement in the understanding of biological function akin to other high-throughput improvements in biology, such as expression arrays [24] and next-generation sequencing [25], and anticipate numerous paradigm-shifting breakthroughs over the coming years.

## Methods

### Training and validation image preparation

Convolutional neural networks using traditional neural network layers for classification can be applied to images of any reasonable size, but once trained at a certain size, this must remain consistent. We chose input sizes of 32 × 32 pixels for root tip images and 64 × 64 pixels for shoot feature images. In the root domain, a 32 × 32 image was found to be adequate to capture a root tip feature, along with enough context from the surrounding image. The 64 × 64 resolution of shoot features was chosen as a compromise between efficiency and the higher resolution necessary to handle the more complex features seen in these images. Choosing a size appropriate to the feature of interest whilst maintaining a balance with computational efficiency is key here.

For root images, we obtained root tip positions from an existing database of manually annotated root systems, paired with the captured input images. For each source image, we created cropped training images centred on each recorded root tip position. This resulted in a variable number of training images per source image, depending on how many root tips had been annotated by the user. We restricted root tip images to primary and lateral roots that were longer than half the window size (16 pixels). Avoiding extremely short lateral roots avoids ambiguity with root hairs, which appear frequently on many of the images. For all training images in the root dataset, we cropped source images at 42 × 42 pixels, and then performed an additional crop to 32 × 32 randomly during training. This approach, known as data augmentation, is akin to producing many more training images with variation in the location of the tips within the cropped windows, such that the root tips do not appear in the exact centre of each training image every time. This approach has been shown to produce improved accuracy when the classification target is not necessarily in the centre of each image, as may be the case when we use our scanning localization approach.

We additionally generated negative training images, which do not contain the features of interest, with two times more negative images than positive ones. We increased the number of negative images in order to adequately capture the wide variety of different negative images that are possible on in this data. Half of the negative data was generated at random points on the source image, but limited to areas that contained no root tips. The remaining negative data were generated at random positions on the known root system, again avoiding root tips. This is a form of hard negative mining, where negative data are generated on regions that appear similar to the positive data. We want the network to learn that we are only interested in tips of roots, not other structures on the root. This has been shown to improve the accuracy of machine learning algorithms over negative data produced entirely at random [26]. The total number of images produced was 43 641, which was split at random into a training set of 34 912 and a validation set of 8729.

A similar approach was used for the preparation of shoot feature images. For each source image, we selected cropped images at each manually annotated location, as with the root tips. The shoot images are higher resolution than the root images, so we found that we obtained better accuracy if we cropped 128 × 128 images, then scaled to 64 × 64 for use in the network. This simply includes more of each image within the field of view of the smaller windows; i.e., we retain more contextual information. Each type of feature (e.g., leaf tip, ear tip) was summed to produce an overall positive image count, and we then generated an equal number of negative images per source image. Unlike the root system data, where information on the position of the remaining root system (derived from the manual annotations) could be used to generate hard negative data, the shoot annotations only included the specific features to be classified. In order to generate hard negative data, we used a Harris feature detector [27] to generate candidate points of interest, then selected from this set at random (discounting areas around positive features). This ensured that the negative data contained large amounts of clutter and other plant material, rather than just plain background regions. Finally, we generated a small number of additional images from truly random locations to ensure that areas such as the white background were represented sufficiently. The resulting dataset contained 62 118 images, of which 49 694 were training images, and the remaining 12 424 were used for validation.

At this point, we have constructed suitable training sets of images derived from manual annotations. The next task is to develop the network architecture itself and train the subsequent networks.

### CNN architecture design

We used the Caffe deep learning library [28] to develop each network. In Caffe, networks are described using a series of structured files, along with information on training and validation, such as how frequently to perform validation when training iterations, and so-called hyperparameters, such as the learning rate, which will be described below.

**Figure 4:**
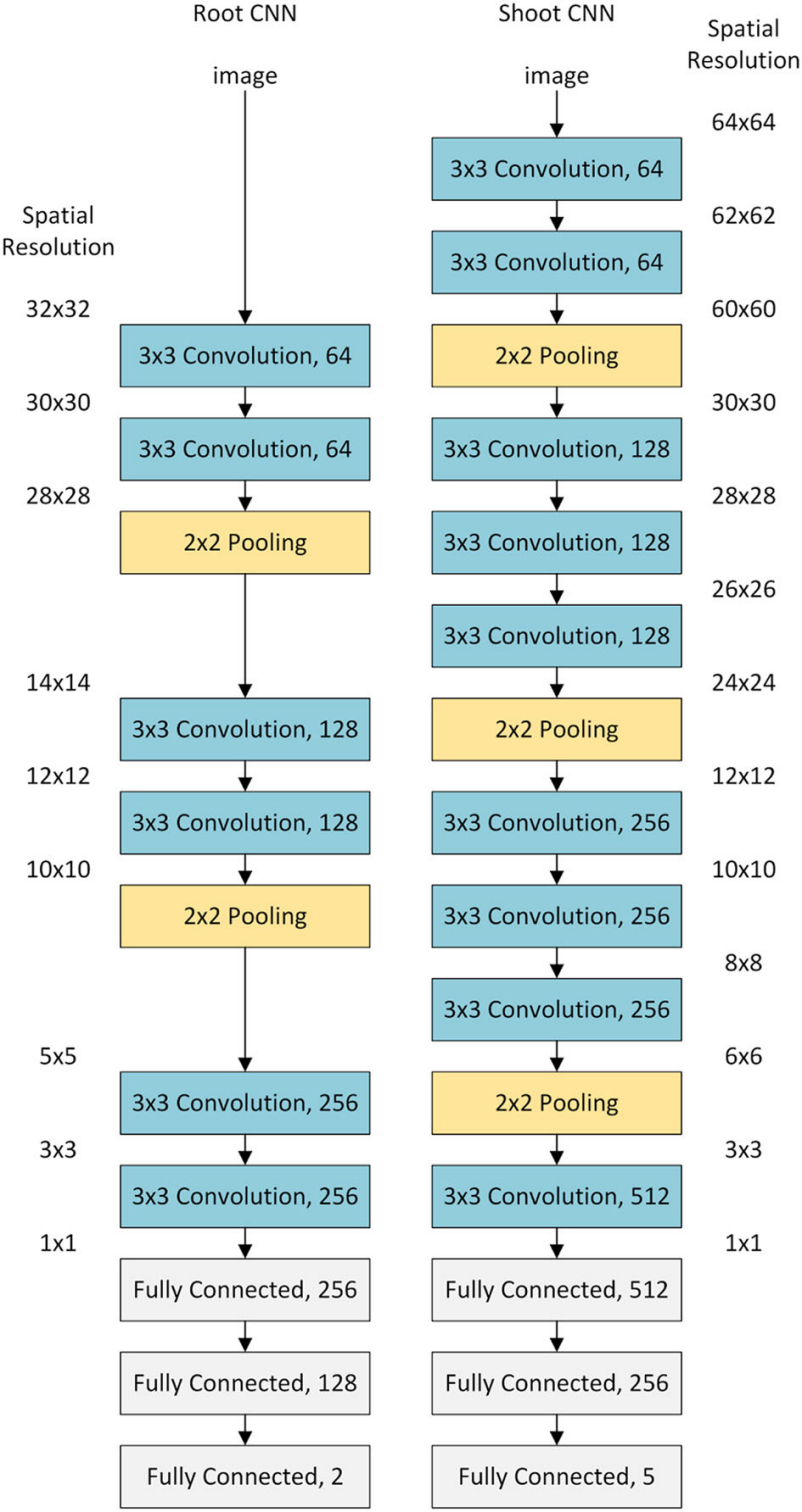
The architecture of both convolutional neural networks (left: root, right: shoot). In each case, convolution and pooling layers reduce the spatial resolution to 1 × 1, while increasing the feature resolution. All convolutional layers used kernels size 3 × 3 pixels, and the number of different filters is shown at the right of each layer. Following the convolution and pooling layers, the fully connected (neural network) layers perform classification of the images. We included rectified linear unit (ReLu) layers between all convolutional and fully connected layers, and dropout layers between each fully connected layer.

We designed separate CNN architectures for each problem. These architectures are shown in Fig. 4; they adopt a common approach to CNN design, utilizing multiple convolutional layers using 3 × 3 kernels prior to each pooling layer [29]. The shoot CNN contains more layers to accommodate the larger input image size. It also includes increased feature counts in deeper layers to address the more challenging classification task posed by the shoot images. Both networks end in neural network classification layers (often referred to as fully connected layers) that reduce the output sizes to 2 and 5, respectively. Once trained, these final neurons represent the likelihood that the network has observed each class (e.g., root tip or not root tip) and can be read to determine which class the network has identified.

The root CNN contained two groups of two convolutional layers, and one max pooling layer. Following these, two final convolutional layers performed further feature extraction, before three standard neural network layers performed the classification. The feature size of the convolutional layers was increased after each pooling layer, beginning at 64 convolutional filters, up to 256 filters. Finally, the neural network layers gradually reduced the feature size back down to two, representing the separate “root tip” and “root negative” classes.

The shoot CNN contains three groups of convolutions and pooling layers. The number of convolutional layers between pooling layers varied slightly throughout the architecture in order to ensure that the spatial resolution of the data was always a multiple of two. A single final convolution is followed by three neural network layers performing the classification. The feature sizes of the convolutional and neural network layers were also increased beyond that of the root CNN. Feature sizes started at 64 filters, up to a maximum of 512 filters. The neural network layers decrease this feature size back down to 5, representing the 5 classes being detected.

Recent developments in CNNs have proposed additional components that improve performance. Neural networks require non-linear functions between layers in order to capture the complex non-linearity of the classification tasks. Traditionally, sigmoid or tanh functions have been used, where the result of each convolutional filter at each position is passed into a nonlinear function before being passed to the next layer. More recent work [10] proposed an alternative function, the rectified linear unit (“Relu”), which has been shown to improve the speed of training deep networks. We utilized Relu layers between all convolutional layers and between all fully connected neural network layers. Other work [30] proposed an approach whereby a percentage of fully connected neurons is randomly deactivated during each iteration of training; this has been shown to avoid the overfitting problem, in which the classification of the training data improves, but at the expense of generality on the unseen data. By deactivating neurons some of the time, the fully connected layers are forced to learn from all parts of the network, rather than become focused on a few key neurons. We included dropout layers with a 50% dropout rate between the fully connected layers.

### CNN training and validation

The Caffe library is built to perform iterative training and validation for as long as is required. Periodically, the accuracy of the networks was measured using the separate validation data, and learning was halted after a steady state was reached, where no further improvement was seen if the network was left training. The learning rate specifies how quickly the network attempts to improve based upon the current set of images it is examining. This is an important feature of network learning; a low learning rate will mean the network does not adapt sufficiently fast to correctly classify the images it sees. A learning rate that is too high may cause the network to wildly over-adapt, meaning it will improve on the current set of images, but at the expense of all the images it has seen previously. As with most modern CNN approaches, we chose a higher learning rate to begin training, then periodically decreased this rate to “refine” the network to higher and higher accuracies. We began with a learning rate of 0.1, then decreased the learning rate by a factor of 10 every 20 000 iterations. In practice, we found that our networks were robust to changes in this learning rate, but that we stopped seeing any real improvement in accuracy when the learning rate fell below 1 × 10^−3^. Before entry into the network, the mean image colour for each dataset was subtracted from each image in order to centre pixel values around 0.

## Availability of data and materials

Data further supporting this work, such as the root and shoot image datasets, as well as the Root Caffe model and Shoot Caffe model, are open and available in the *GigaScience* repository, *Giga*DB [12]. Further details on the methods used in this study are also available at Protocols.io [31].

## Additional files

Additional file 1: Images showing the response of our classifier using a sliding window over selected input images, for roots and shoots. Roots: regions of high response from the classifier are shown in yellow. Shoots: regions of high response from the classifier for leaf tips are highlighted in orange, leaf bases in yellow, ear tips in blue, ear bases in pink.

Additional file 2: Unseen test images. Pairs of images are presented: original images on the left, localized features shown on the right. Roots: regions of high response from the classifier are shown in yellow. Shoots: regions of high response from the classifier for leaf tips are highlighted in orange, leaf bases in yellow, ear tips in blue, ear bases in pink.

Additional file 3: Confusion matrices for the root and shoot classification datasets.

## Abbreviations

CNN: Convolutional Neural Network; QTL: Quantitative Trait Loci.

## Competing interests

The authors declare that they have no competing interests.

## Funding

This work was supported by the Biotechnology and Biological Sciences Research Council [grant number BB/N018575/1], partially funding APF and MPP; and a European Research Council Advanced Grant [FUTUREROOTS 294729], funding JAA.

## Author contributions

M.P.P. developed the deep learning system and image processing and carried out the method development, along with A.P.F., D.M.W., and T.P.P. J.A.A. assisted, and collected and annotated data along with A.J.B. and M.G. M.H.W. and E.H.M. assisted with the preparation of the root and shoot datasets, respectively. A.S.J., A.B., and G.T. provided valuable deep learning expertise. A.P.F., M.P.P., D.M.W., J.A.A., and T.P.P. wrote the manuscript, with assistance from all authors.

## Supplementary Material

GIGA-D-17-00122_Original-Submission.pdf

GIGA-D-17-00122_Revision-1.pdf

Response-to-Reviewer-Comments_Original-Submission.pdf

Reviewer-1-Report-(Original-Submission).pdf

Reviewer-2-Report-(Original-Submission).pdf

Reviewer-2-Report-(Revision-1).pdf

Additional files
