# Erratum to: Deep machine learning provides state-of-the-art performance in image-based plant phenotyping

**DOI:** 10.1093/gigascience/giy042

**Published:** 2018-07-20

**Authors:** Michael P Pound, Jonathan A Atkinson, Alexandra J Townsend, Michael H Wilson, Marcus Griffiths, Aaron S Jackson, Adrian Bulat, Georgios Tzimiropoulos, Darren M Wells, Erik H Murchie, Tony P Pridmore, Andrew P French

In the final publication of "Deep machine learning provides state-of-the-art performance in image-based plant phenotyping,” by Michael Poundet al., the publication month for reference 21 was incorrect.

Reference 21 has been corrected and the corrected manuscript is published in *GigaScience*, Volume 6, Issue 10: https://doi.org/10.1093/gigascience/gix083.
