# Supplementary figures and images for: Deep machine learning provides state-of-the-art performance in image-based plant phenotyping
Source: Gigascience. 2017 Aug 23;6(10):1–10. doi: 10.1093/gigascience/gix083 (PMC5632296; doi:10.1093/gigascience/gix083)

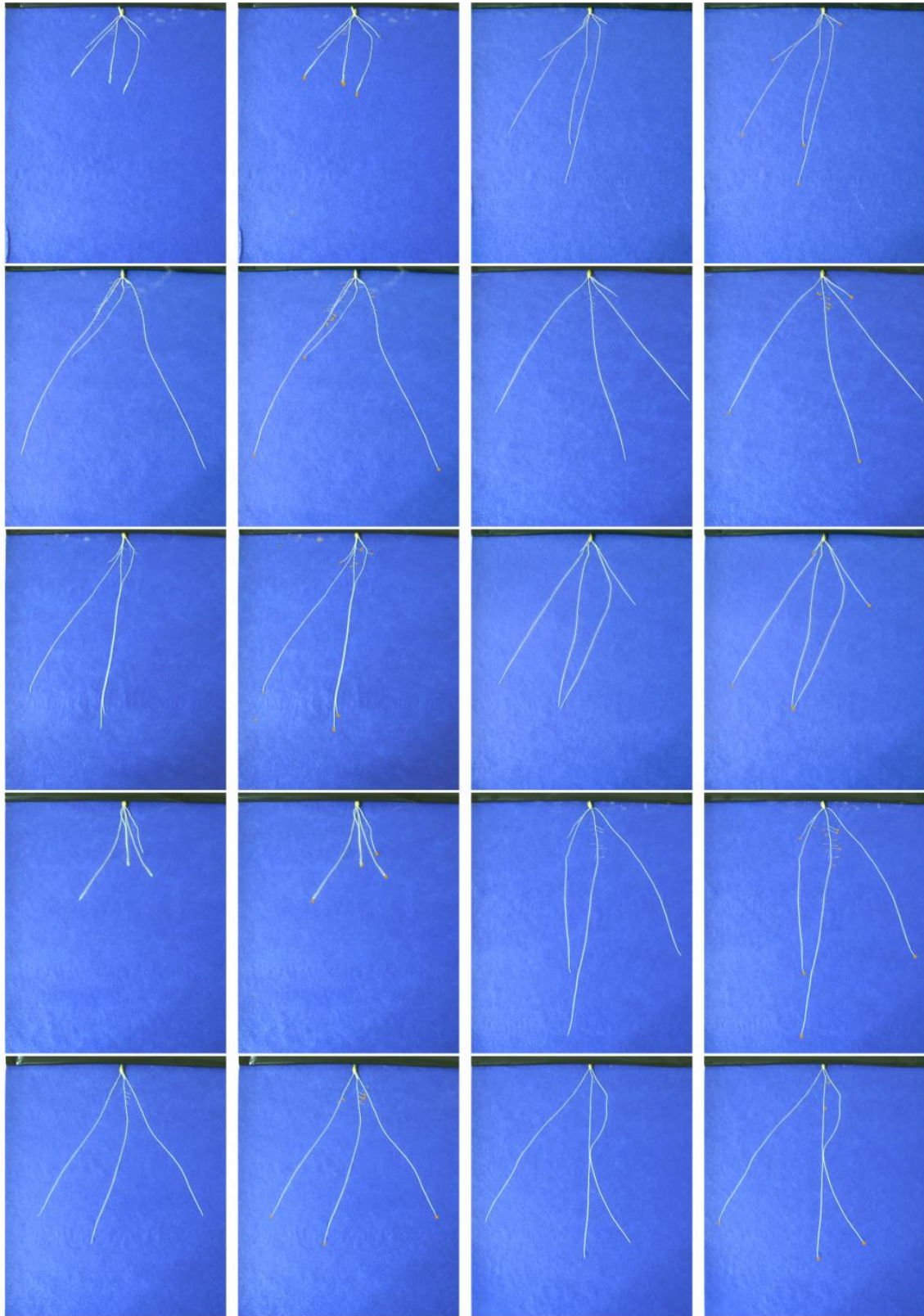

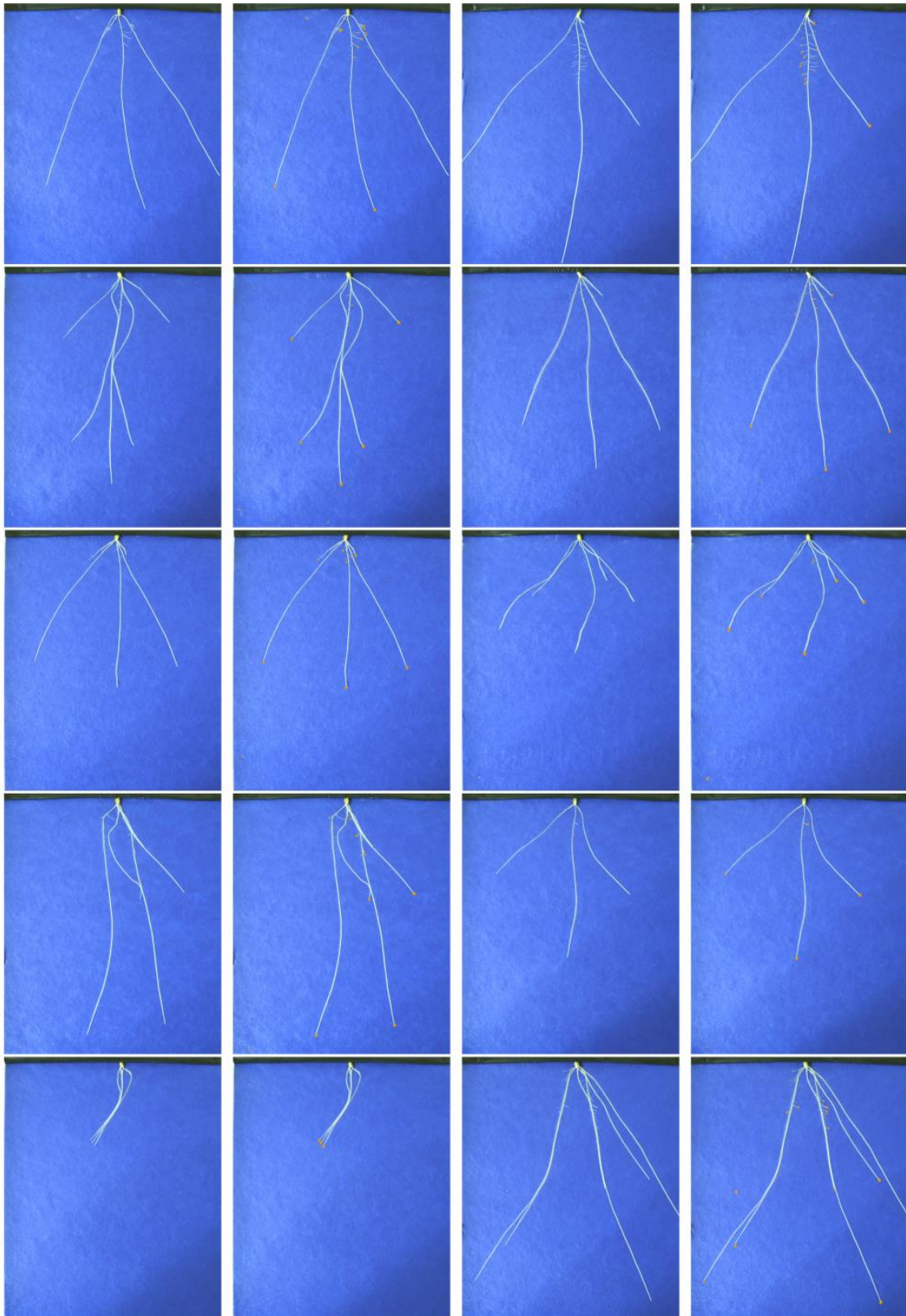

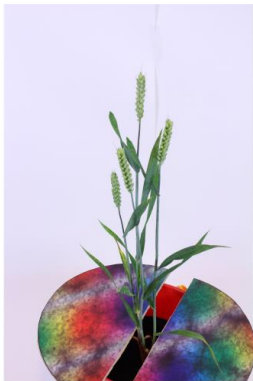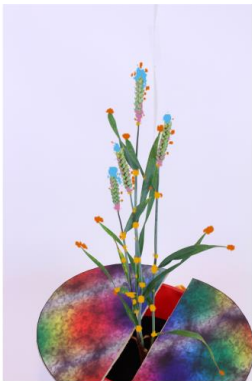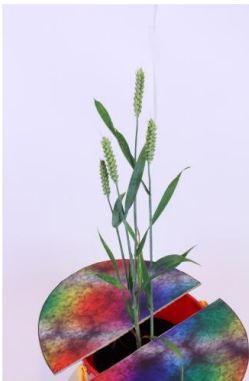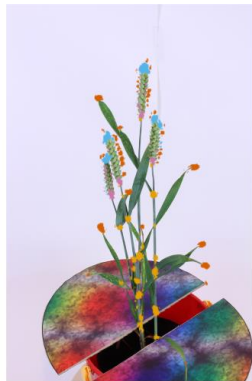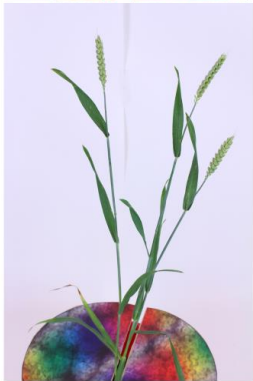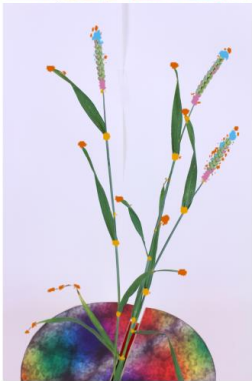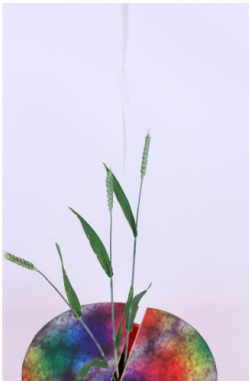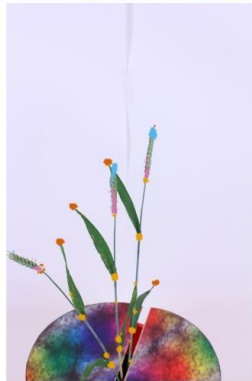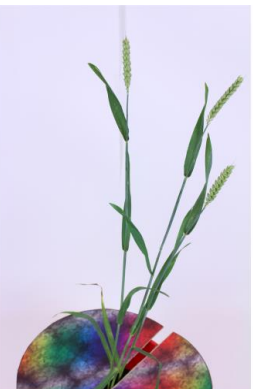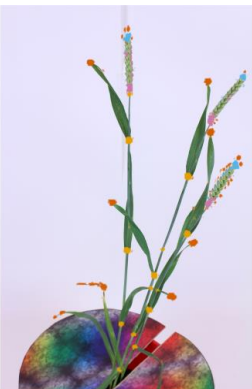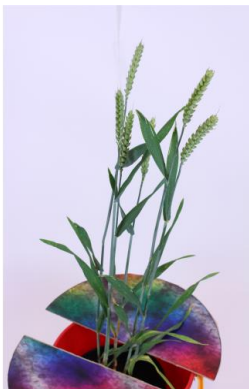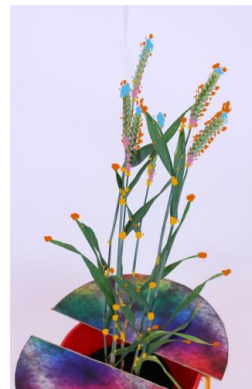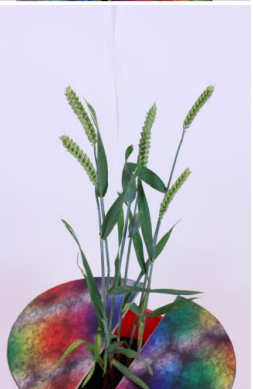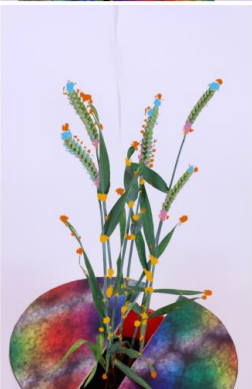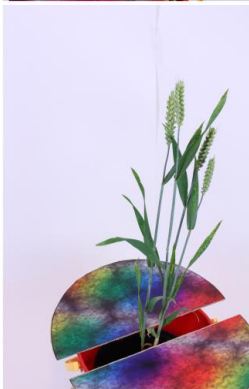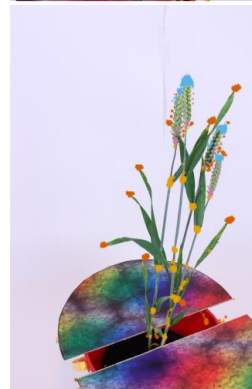

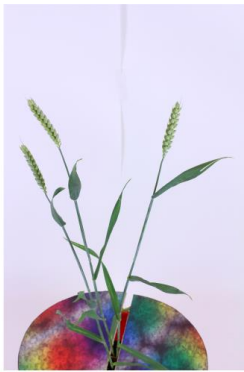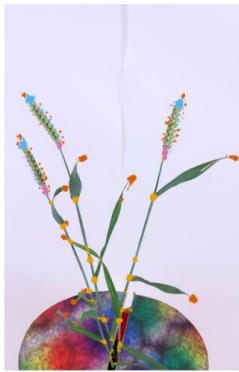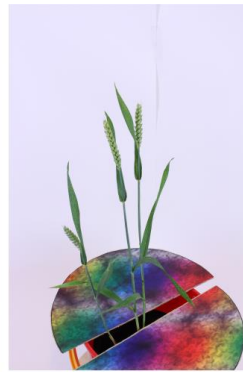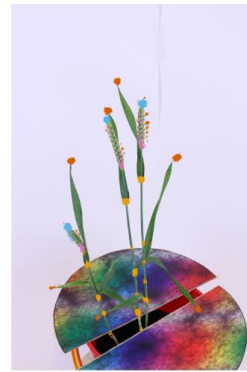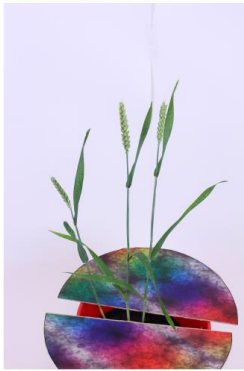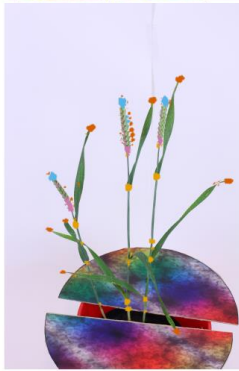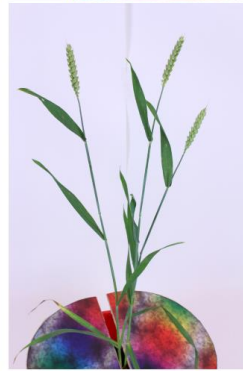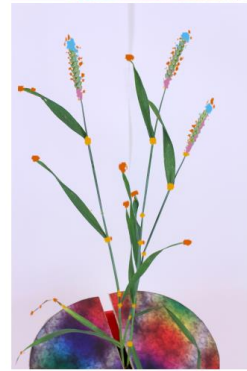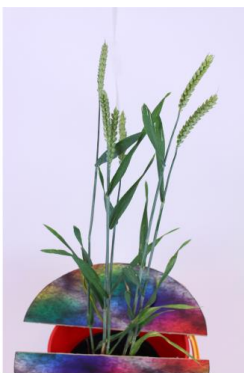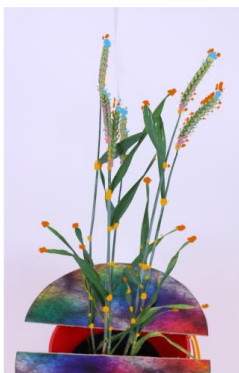

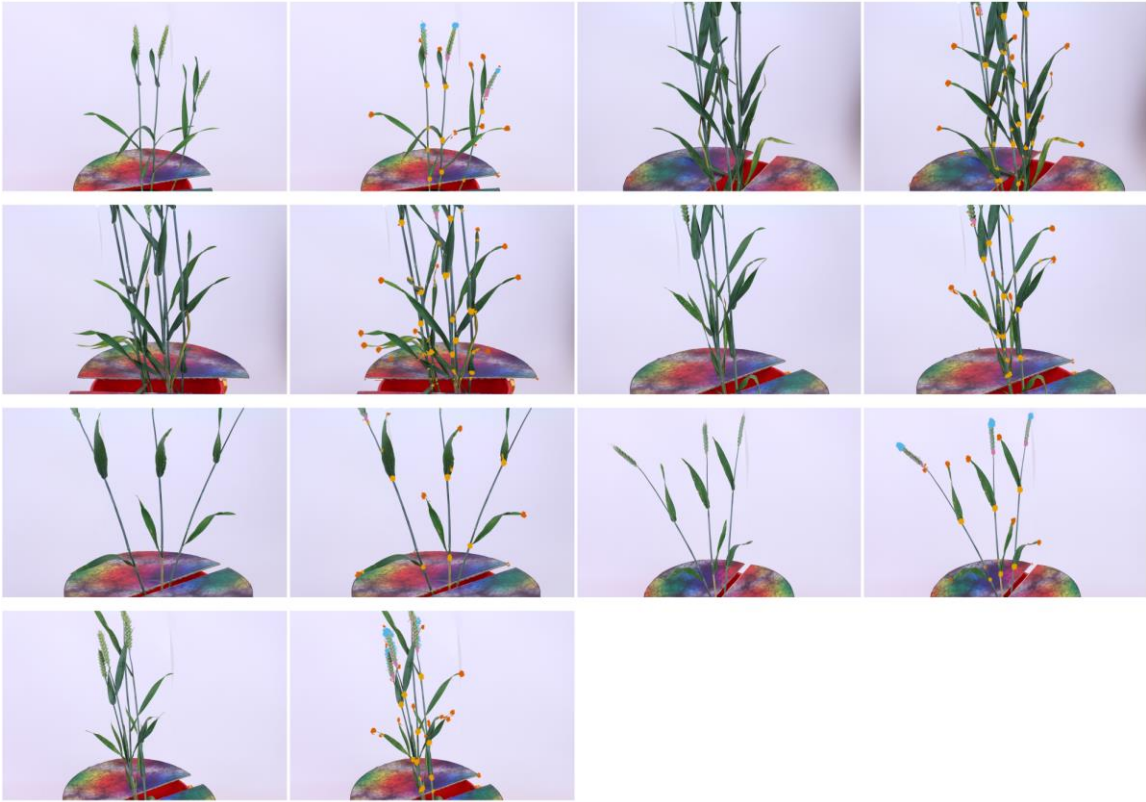

Supplement: Additional files [file gix083_Supp.zip › Additional File 2.pdf]

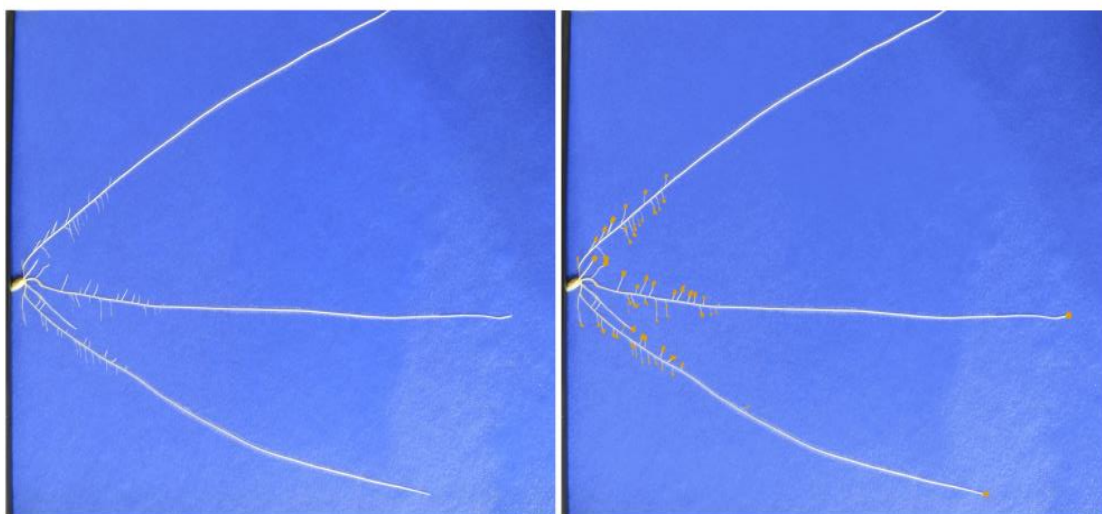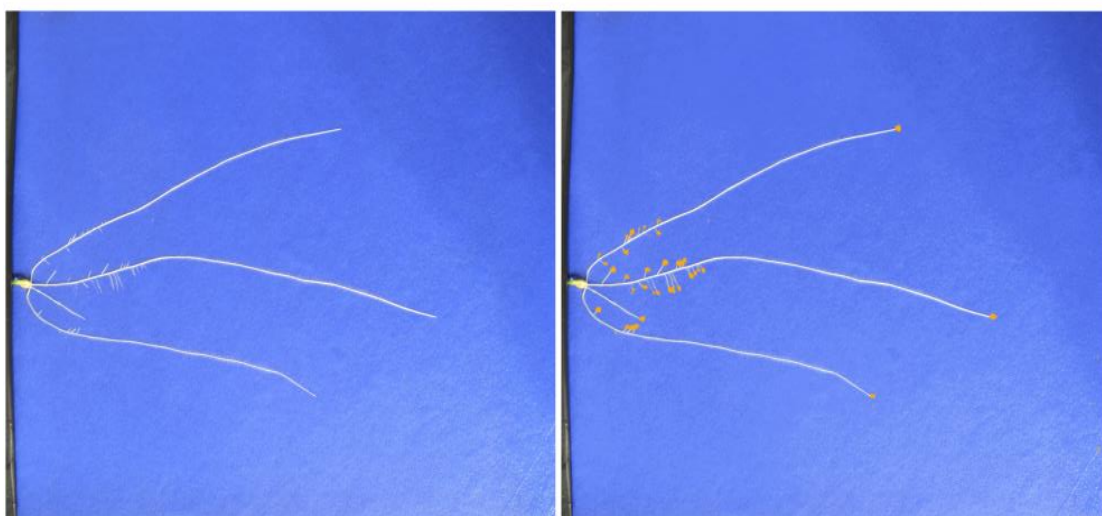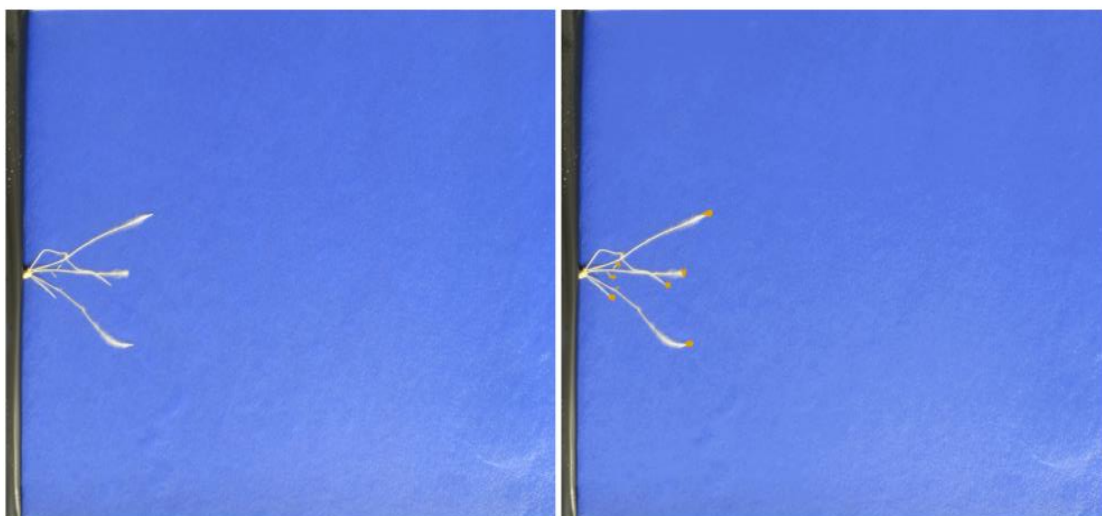

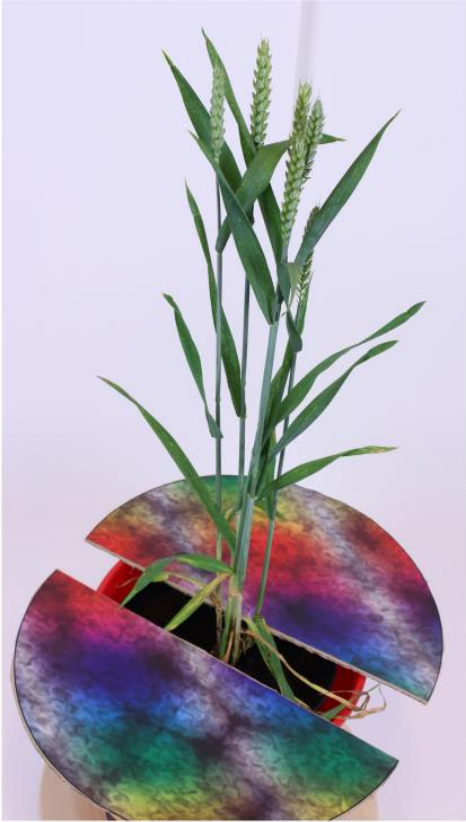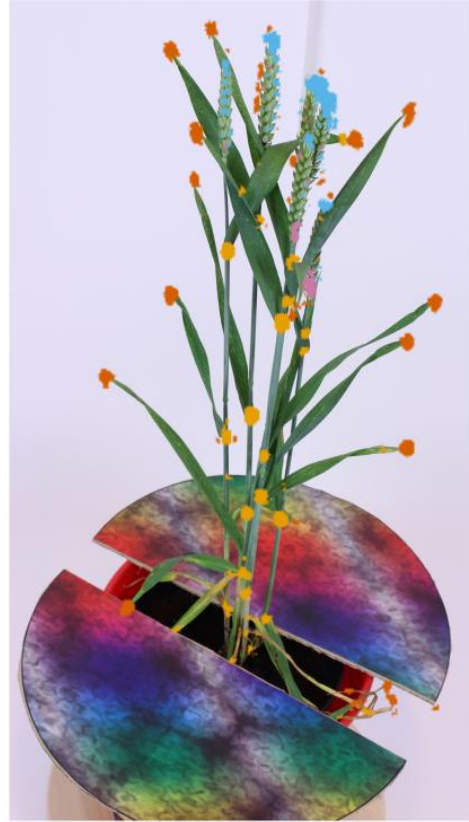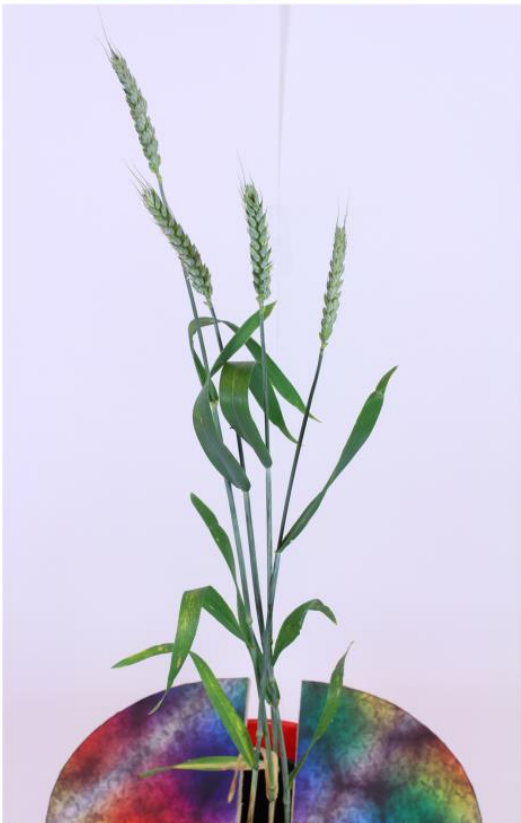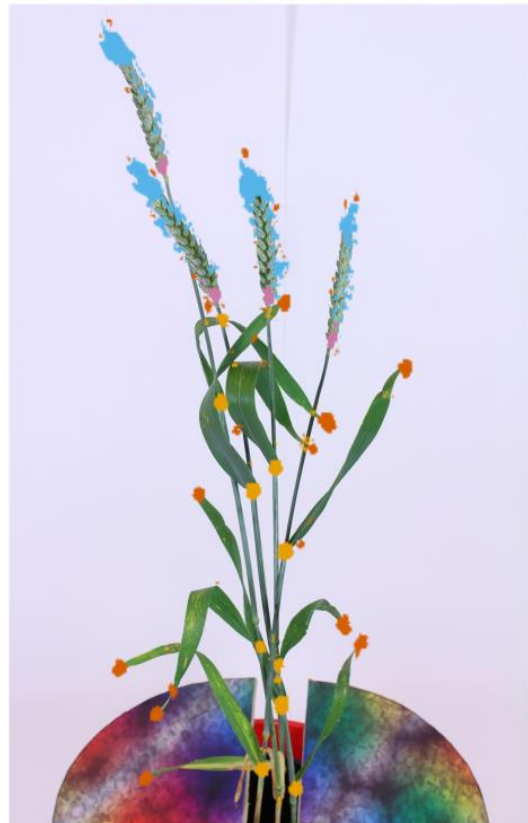

Supplement: Additional files [file gix083_Supp.zip › Additonal File 1.pdf]
