# Supplementary material for: Deep machine learning provides state-of-the-art performance in image-based plant phenotyping
Source: Gigascience. 2017 Aug 23;6(10):1–10. doi: 10.1093/gigascience/gix083 (PMC5632296; doi:10.1093/gigascience/gix083)
Supplement: Response-to-Reviewer-Comments_Original-Submission.pdf [file gix083_Response-to-Reviewer-Comments_Original-Submission.pdf]

Author's Response To Reviewer Comments

[Please see the cover letter for a formatted list of comments, and Additional File 4 for a tracked changes manuscript.]

Reviewer #1: In their paper, Michael Pound and co-workers evaluate the performance (accuracy) of deep machine learning (convolutional neural networks) in plant image analysis for the automated identification and localization of root and shoot features (root tips, leaf tips, leaf bases, ear tips, ear bases). It is worth noting that their approach correctly classify/locate plant features more than 97% of the time. Focusing on a dataset of root images, they also demonstrate that their approach can be used to extract root system traits (based on root tip positions) and identify quantitative trait loci (QTL) in genetic research. In my opinion, a timely and very nice piece of work!

Overall, the manuscript is very well written and pleasant to read. The figures and tables are clear and necessary. I think that this paper is of high interest to the scientific community (not only plant scientists) and could be a valuable contribution to GigaScience because it shows the potential of deep machine learning for the development of automated, accurate, and high-throughput image analysis pipelines. I have only minor comments for this manuscript.

...Many thanks for this very positive review

Minor comments
When I read Table 3, I wondered how the estimation of the total root system length (based on root tip locations) correlates with the ground truth length calculated with RootNav. Based on the description provided in the paper (the sum of the distances from each tip to the seed position), it seems to me that the CNN-derived length might overestimate the true root system length (particularly because I expect the distance between the seed position and a lateral root tip to be greater than the true lateral root length).

...This is intended to be a ‘fast and rough’ estimate of length - with no root segmentation it is all we can do. Overestimation (in fact, probably underestimation) is likely but for the purpose we use here, and as demonstrated by the QTL discovery, it works adequately.

I would try to define an abbreviation each time it appears for the first time. Quantitative trait loci (QTL) in the background section (p. 6, line 40), rectified linear unit (ReLu) in Figure 4's caption.

...We have made these changes, thank you.

P. 14, lines 11-14: you wrote "The QTL for one trait, Centre of Mass (x), was not detected using the deep learning approach, but was found using trait values from RootNav". This suggests that 1 QTL was not detected. Looking at table 4, it seems that the RootNav approach identifies 3 QTL based on Centre of Mass (x), but 2 QTL were not detected using the CNN-derived Centre of Mass (x).

...Thank you for the correction, we have adjusted the text and added a further explanation.

“Finally, it is worth noting though that a second QTL for the same trait was detected on chromosome 6D using both systems.”

P. 15, line 47: where do the 92% come from? If the CNN approach found 12 QTL out of 14 (as written in the abstract), would it not be better to write that the CNN-derived tip detection pipeline successfully found 85.7% (12/14*100) of the tip-related QTL?

...Apologies, 12 out of 14 was the correct figure and we have updated the %

Some keyboard typos

P. 6, line 58: space missing between "previously" and "[12]".
P. 7, line 7: space missing between "RootNav software" and "[13]".
P. 12, line 53: space missing between "images" and "[12]".
P. 14, line 11: "Centre of Mass (x)" instead of "Centre of Max (x)"?
In table 4: "Centre of Mass (y)" instead of "Mentre of Mass (y)"?
Table 4's caption: "CNN-derived approaches" instead of "CNN-derived and approaches"?
P. 20, line 40: "shoot CNN" instead of "root CNN"?

...Many thanks for bringing these to our attention. We have corrected all the typing errors. The last issue was correctly “root CNN” but we have adjusted the sentence to be more clear.

Reviewer #2: This paper deploys deep learning algorithms to learn root and shoot features in wheat and subsequently demonstrate a potential application of this pipeline on QTL mapping through results comparison of manual versus automated phenotyping results.
Overall, these approaches are the latest developments in the multi-disciplinary space and therefore are exciting for the specialists working in this area as well as a wider general audience interested in phenomics.
Please see suggestions to improve this submission:
...Thank you for your time writing this positive and constructive review, and for the specific suggestions below.

1. Background or General Introduction: This section is too long at almost 4 pages (compared to ½ page of discussion section) and has a 'review article' feel to it as excessive information is devoted to explain deep learning neural networks. It will be more relevant to devote some more space to the need for this approach (for root and shoot phenotyping) and current state of the art for these traits (there are several worthwhile studies on these traits that have utilized hand crafted features, as well as supervised and unsupervised learning) and include GWAS and conventional QTL mapping. Include some pertinent references. This will help set the context better rather than reading like a 'Methods' paper. Please note that reviewer duly notes that QTL was only done on root images and not on shoot images (traits).

...Thank you for the suggestion. We feel a lengthy introduction to deep learning is appropriate, as

that is the new development we are highlighting; we feel it will provide useful background information to interested readers, and can be skipped by the reader if necessary.
We have, though, now added more background information on QTL to the introduction.
We do not yet have QTL information with which to compare for the shoot data, and this falls outside the scope of what is possible with the current paper, but is certainly a good suggestion for future work.

2. Page 5, line 42 "…….often falls short of capturing the final 10% of accuracy required for fully automated systems." This value needs to be substantiated with a well-established reference or domain expertise, else there is a fear of this number being misconstrued by other researcher that 90 or above accuracy is the domain expert established level. Please note that different scenarios, including trait type, classification vs quantification, crop species, importance features, biology and economics among other factors determine the optimum cutoff level. If the authors wish to keep 90% accuracy as their opinion, a qualifying statement needs to be included to clarify this conundrum.

...Many thanks for pointing this out, you are absolutely right. We have rephrased this sentence, removing the reference to this value to avoid confusion.

3. The first classification problem ("…..given a small section of a root system image, can a CNN identify if a root tip is present?") implies that the work only consisted of a yes/no classification; however, in Table 3 several root traits are listed. This needs to be addressed and clarified in the classification problem and CNNs subsequent use so readers are able to better grasp the scope of work.

...We have clarified the scope of the work and the subsequent generation of traits from the CNN in the introduction.

4. Data description section: Previous studies have been cited for phenotyping data (manual - root and shoot) but it is extremely distracting to navigate between different papers (current and previous QTL papers); therefore, please include more information so that this submission is 'stand-alone'.

...Whilst we recognise this introduces an extra step of indirection, this is out of necessity standard practice in biological papers, as the protocols for acquiring the data and using the software can be lengthy. However, we have added in more high-level information about the procedures into the section to hopefully add clarity.

5. Include image pre-processing and outlier removal (procedure, justification), if done prior to deploying CNN.

...In fact, we performed no outlier removal or image pre-processing with a view to altering image quality. Before inputting each image into the network, the mean image colour was subtracted from all pixels, in order to center the data around zero (standard for CNN use). We have added a sentence explaining this in the training section of the methods.

6. In Table 1, if you are referring to 'Root Negative' from negative training images, please include a footnote to clarify it in table itself. If this is not the case, please clarify what root negative means.

...Thanks for pointing this out, this was unclear. We have altered the label in this table to refer to “Root Tip Negative” which is what was the intended meaning.

7. Table, it will be useful to see the complete confusion matrix to determine % of all four classes.

...We thank the reviewer for this suggestion, a confusion matrix would make a useful addition. We have added confusion matrices for both root and shoot classification in the supplementary materials.

8. These accuracies are dependent on root imaging software (assumed) as the ground truth. What were the % of manual misclassification and variability associated with it (Including mean per class accuracy)? Cross validation will be helpful to assess accuracies and applicability in unforeseen data.

...Thank you for highlighting the important point of annotation accuracy. Whilst we have not calculated these measures in this work, we have added a paragraph to the discussion to raise awareness of this. Here, identifying the same QTLs as previous work is sufficient, but certainly this is an important concept which should not be omitted.

9. Root tip trait is listed as a bottleneck in phenotyping, why?

...We suggest, based on [13], that root tip localisation is a bottleneck specifically for automated systems. In [13] and numerous other software systems (e.g. Smartroot, RootTrace) analysis of root traits is almost fully automatic after an initial user intervention to determine a tip or start location. We have added a reference to the text to help clarify this.

10. In Table 3, following traits are listed: Tip Count, Hull area, Width / Depth, Width:Depth Ratio, Mean X / Y, Standard Deviation X / Y, Top 100 / 200 / 300px count, Total Length, Centre Mass X / Y; while in Table 4, several dissimilar traits (compared to Tabel 3 list) are presented. These include: Centre of Mass (X) compared to Centre Mass (x/y) in table 3; Total root length compared to Total length in table 3; Convex Hull compared to Hull area in table 3; Lateral count/ Tip Count which is missing in table 3; Maximum Depth which is missing in Table 3; Maximum Width which is missing in Table 3. This needs careful proofing and only relevant and correct information should be included. Where applicable, trait unit needs to be listed. Also, proof if Mentre of Mass (y) in Table 4 should be Centre of Mass (y).

...We believe there may be a misunderstanding here, which we have hopefully now clarified. The traits descriptions from Table 3 and 4 are derived from different sources, so we expect them to be different. Table 3 shows artificial estimated traits derived from the CNN output; table 4 shows traits previously determined in prior QTL analysis.

11. Is possible, please increase the testing set (currently at 20 images each for root and shoot) as these numbers are too low and re-do the analysis. If you do not have access to this data, please address it in discussion section by displaying caution on lower # of testing set.

...To be clear, this is an extra image set “from the wild”; completely unseen by any CNN training algorithms. The aim was to demonstrate that the network really does generalise to new data. The final unseen testing images demonstrate that we have not overfit to the validation set, for example by stopping training at some anomalous peak in validation accuracy. We feel the results on the testing set demonstrate the generality of the approach. Given the substantial validation and testing already performed, we feel it is unlikely that additional testing images will bring new insight to our results.

12. Please format Table 2, so that term 'Shoots' in column 1 aligns with 'Leaf tip' in column 2. How was Total accuracy (%) obtained for shoots. 99.07% is lower than each of the four shoot traits.

...Thanks for pointing out the discrepancy here. The issue here was that feature accuracy was averaged, whereas total accuracy was summed over all features. We agree this is a confusing way of presenting this data, so have removed the “Total Accuracy” measure from the table. These results are still included in the text below table 2, and have been further explained with additional text. We hope this is more clear.

13. Table 4: Arrange table on traits, not chromosomes.

...Whist we agree arranging the table by traits could be seen as more logical, the convention when publishing QTL data is to group by chromosome (as we did in the original research paper). To enable easy comparison with Table 2 in the original paper, we have left the organisation of the table as-is. We have updated the contents though, as per point 14 below.

14. Table 4: Include marker name. Also, details (software, parameters, analysis type, conditions) on how QTL analysis was performed needs to be provided.

...Nearest marker names for the deep learning-derived QTL have been added to Table 4. As stated in the text, the QTL analysis followed exactly the same pipeline as detailed in the Atkinson et al. (2015) paper. We have added additional text in the section to re-iterate this information for clarity.

15. Table 4: why do you see difference in LOD scores between manual and deep learning obtained features? You have obtained extremely high accuracies (please ensure your model is not over-fitting), but report large variation in LOD scores. An explanation of why this can happen needs to be included in the discussion section. Please also include the additive effect of each QTL in this Table.

...Differences in the LOD score for comparable QTL between the deep learning and RootNav approaches are most likely due to differences in how the traits values are calculated in each approach. For deep learning, trait values were estimated based on tip positions which will

produce results comparable to (but not identical to) those derived from RootNav, particularly for length based traits. Furthermore, any missed root tips would cause subtle differences to trait values altering the BLUP values then used for QTL discovery. Combined, these differences in trait values across wheat genotypes will alter the LOD values and positions of the QTL discovered. Additive effects of each QTL have been added to the table.

16. Make table captions stand alone. Needs to be further populated.
...Thanks for the advice - we have added more detail to some of the captions.

17. Discussion requires substantial work. Authors are urged to improve this section substantially. This needs to help tie in the background information you provide with the outputs of this research. Highlight the main findings and relate to similar studies. There needs to be a detailed explanation of interpretation of your findings, and does it agree /not agree with previous work highlighting the power of DL. Need to include relevant references, and also challenges or limitations of this work as well as further research.

...We have added more paragraphs to the discussion, including: a discussion of the importance of and limitations of annotation quality, a further look at future work, and an additional DL reference with which to compare.
