# Supplementary material for: Deep machine learning provides state-of-the-art performance in image-based plant phenotyping
Source: Gigascience. 2017 Aug 23;6(10):1–10. doi: 10.1093/gigascience/gix083 (PMC5632296; doi:10.1093/gigascience/gix083)
Supplement: Reviewer-1-Report-(Original-Submission).pdf [file gix083_Reviewer-1-Report-(Original-Submission).pdf]

Reviewer Report

Title: Deep Machine Learning provides state-of-the-art performance in image-based plant phenotyping

Version: Original Submission **Date:** 6/8/2017

Reviewer name: Benjamin Delory

Reviewer Comments to Author:

In their paper, Michael Pound and co-workers evaluate the performance (accuracy) of deep machine learning (convolutional neural networks) in plant image analysis for the automated identification and localization of root and shoot features (root tips, leaf tips, leaf bases, ear tips, ear bases). It is worth noting that their approach correctly classify/locate plant features more than 97% of the time. Focusing on a dataset of root images, they also demonstrate that their approach can be used to extract root system traits (based on root tip positions) and identify quantitative trait loci (QTL) in genetic research. In my opinion, a timely and very nice piece of work!
Overall, the manuscript is very well written and pleasant to read. The figures and tables are clear and necessary. I think that this paper is of high interest to the scientific community (not only plant scientists) and could be a valuable contribution to GigaScience because it shows the potential of deep machine learning for the development of automated, accurate, and high-throughput image analysis pipelines. I have only minor comments for this manuscript.
Minor comments
When I read Table 3, I wondered how the estimation of the total root system length (based on root tip locations) correlates with the ground truth length calculated with RootNav. Based on the description provided in the paper (the sum of the distances from each tip to the seed position), it seems to me that the CNN-derived length might overestimate the true root system length (particularly because I expect the distance between the seed position and a lateral root tip to be greater than the true lateral root length).
I would try to define an abbreviation each time it appears for the first time. Quantitative trait loci (QTL) in the background section (p. 6, line 40), rectified linear unit (ReLu) in Figure 4's caption.
P. 14, lines 11-14: you wrote "The QTL for one trait, Centre of Mass (x), was not detected using the deep learning approach, but was found using trait values from RootNav". This suggests that 1 QTL was not detected. Looking at table 4, it seems that the RootNav approach identifies 3 QTL based on Centre of Mass (x), but 2 QTL were not detected using the CNN-derived Centre of Mass (x).
P. 15, line 47: where do the 92% come from? If the CNN approach found 12 QTL out of 14 (as written in the abstract), would it not be better to write that the CNN-derived tip detection pipeline successfully found 85.7% (12/14*100) of the tip-related QTL?
Some keyboard typos
P. 6, line 58: space missing between "previously" and "[12]".
P. 7, line 7: space missing between "RootNav software" and "[13]".
P. 12, line 53: space missing between "images" and "[12]".
P. 14, line 11: "Centre of Mass (x)" instead of "Centre of Max (x)"?

In table 4: "Centre of Mass (y)" instead of "Mentre of Mass (y)"?
Table 4's caption: "CNN-derived approaches" instead of "CNN-derived and approaches"?
P. 20, line 40: "shoot CNN" instead of "root CNN"?

Methods

Are the methods appropriate to the aims of the study, are they well described, and are necessary controls included? Yes

Conclusions

Are the conclusions adequately supported by the data shown? Yes

Reporting Standards

Does the manuscript adhere to the journal's guidelines on [minimum standards of reporting?]() YesChoose an item.

Statistics

Are you able to assess all statistics in the manuscript, including the appropriateness of statistical tests used? No, and I do not feel adequately qualified to assess the statistics.

Quality of Written English

Please indicate the quality of language in the manuscript: Acceptable

Declaration of Competing Interests

Please complete a declaration of competing interests, considering the following questions:

- Have you in the past five years received reimbursements, fees, funding, or salary from an organisation that may in any way gain or lose financially from the publication of this manuscript, either now or in the future?
- Do you hold any stocks or shares in an organisation that may in any way gain or lose financially from the publication of this manuscript, either now or in the future?
- Do you hold or are you currently applying for any patents relating to the content of the manuscript?
- Have you received reimbursements, fees, funding, or salary from an organization that holds or has applied for patents relating to the content of the manuscript?
- Do you have any other financial competing interests?
- Do you have any non-financial competing interests in relation to this paper?

If you can answer no to all of the above, write 'I declare that I have no competing interests' below. If your reply is yes to any, please give details below.

I declare that I have no competing interests

I agree to the open peer review policy of the journal. I understand that my name will be included on my report to the authors and, if the manuscript is accepted for publication, my named report including any attachments I upload will be posted on the website along with the authors' responses. I agree for my report to be made available under an Open Access Creative Commons CC-BY license (http://creativecommons.org/licenses/by/4.0/). I understand that any comments which I do not wish to be included in my named report can be included as confidential comments to the editors, which will not be published.

I agree to the open peer review policy of the journal

To further support our reviewers, we have joined with Publons, where you can gain additional credit to further highlight your hard work (see: https://publons.com/journal/530/gigascience). On publication of this paper, your review will be automatically added to Publons, you can then choose whether or not to claim your Publons credit. I understand this statement.

Yes
