# Supplementary material for: Deep machine learning provides state-of-the-art performance in image-based plant phenotyping
Source: Gigascience. 2017 Aug 23;6(10):1–10. doi: 10.1093/gigascience/gix083 (PMC5632296; doi:10.1093/gigascience/gix083)
Supplement: Reviewer-2-Report-(Original-Submission).pdf [file gix083_Reviewer-2-Report-(Original-Submission).pdf]

Reviewer Report

Title: Deep Machine Learning provides state-of-the-art performance in image-based plant phenotyping

Version: Original Submission **Date:** 7/18/2017

Reviewer name: Asheesh Singh

Reviewer Comments to Author:

This paper deploys deep learning algorithms to learn root and shoot features in wheat and subsequently demonstrate a potential application of this pipeline on QTL mapping through results comparison of manual versus automated phenotyping results.
Overall, these approaches are the latest developments in the multi-disciplinary space and therefore are exciting for the specialists working in this area as well as a wider general audience interested in phenomics.
Please see suggestions to improve this submission:
1. Background or General Introduction: This section is too long at almost 4 pages (compared to ½ page of discussion section) and has a 'review article' feel to it as excessive information is devoted to explain deep learning neural networks. It will be more relevant to devote some more space to the need for this approach (for root and shoot phenotyping) and current state of the art for these traits (there are several worthwhile studies on these traits that have utilized hand crafted features, as well as supervised and unsupervised learning) and include GWAS and conventional QTL mapping. Include some pertinent references. This will help set the context better rather than reading like a 'Methods' paper. Please note that reviewer duly notes that QTL was only done on root images and not on shoot images (traits).
2. Page 5, line 42 "…….often falls short of capturing the final 10% of accuracy required for fully automated systems." This value needs to be substantiated with a well-established reference or domain expertise, else there is a fear of this number being misconstrued by other researcher that 90 or above accuracy is the domain expert established level. Please note that different scenarios, including trait type, classification vs quantification, crop species, importance features, biology and economics among other factors determine the optimum cutoff level. If the authors wish to keep 90% accuracy as their opinion, a qualifying statement needs to be included to clarify this conundrum.
3. The first classification problem ("…..given a small section of a root system image, can a CNN identify if a root tip is present?") implies that the work only consisted of a yes/no classification; however, in Table 3 several root traits are listed. This needs to be addressed and clarified in the classification problem and CNNs subsequent use so readers are able to better grasp the scope of work.
4. Data description section: Previous studies have been cited for phenotyping data (manual - root and shoot) but it is extremely distracting to navigate between different papers (current and previous QTL papers); therefore, please include more information so that this submission is 'stand-alone'.
5. Include image pre-processing and outlier removal (procedure, justification), if done prior to deploying CNN.
6. In Table 1, if you are referring to 'Root Negative' from negative training images, please include a footnote to clarify it in table itself. If this is not the case, please clarify what root negative means.

7. Table, it will be useful to see the complete confusion matrix to determine % of all four classes.
8. These accuracies are dependent on root imaging software (assumed) as the ground truth. What were the % of manual misclassification and variability associated with it (Including mean per class accuracy)? Cross validation will be helpful to assess accuracies and applicability in unforeseen data.
9. Root tip trait is listed as a bottleneck in phenotyping, why?
10. In Table 3, following traits are listed: Tip Count, Hull area, Width / Depth, Width:Depth Ratio, Mean X / Y, Standard Deviation X / Y, Top 100 / 200 / 300px count, Total Length, Centre Mass X / Y; while in Table 4, several dissimilar traits (compared to Tabel 3 list) are presented. These include: Centre of Mass (X) compared to Centre Mass (x/y) in table 3; Total root length compared to Total length in table 3; Convex Hull compared to Hull area in table 3; Lateral count/ Tip Count which is missing in table 3; Maximum Depth which is missing in Table 3; Maximum Width which is missing in Table 3. This needs careful proofing and only relevant and correct information should be included. Where applicable, trait unit needs to be listed. Also, proof if Mentre of Mass (y) in Table 4 should be Centre of Mass (y).
11. Is possible, please increase the testing set (currently at 20 images each for root and shoot) as these numbers are too low and re-do the analysis. If you do not have access to this data, please address it in discussion section by displaying caution on lower # of testing set.
12. Please format Table 2, so that term 'Shoots' in column 1 aligns with 'Leaf tip' in column 2. How was Total accuracy (%) obtained for shoots. 99.07% is lower than each o the four shoot traits.
13. Table 4: Arrange table on traits, not chromosomes.
14. Table 4: Include marker name. Also, details (software, parameters, analysis type, conditions) on how QTL analysis was performed needs to be provided.
15. Table 4: why do you see difference in LOD scores between manual and deep learning obtained features? You have obtained extremely high accuracies (please ensure your model is not over-fitting), but report large variation in LOD scores. An explanation of why this can happen needs to be included in the discussion section. Please also include the additive effect of each QTL in this Table.
16. Make table captions stand alone. Needs to be further populated.
17. Discussion requires substantial work. Authors are urged to improve this section substantially. This needs to help tie in the background information you provide with the outputs of this research. Highlight the main findings and relate to similar studies. There needs to be a detailed explanation of interpretation of your findings, and does it agree /not agree with previous work highlighting the power of DL. Need to include relevant references, and also challenges or limitations of this work as well as further research.

Methods

Are the methods appropriate to the aims of the study, are they well described, and are necessary controls included? Yes

Conclusions

Are the conclusions adequately supported by the data shown? Yes

Reporting Standards

Does the manuscript adhere to the journal's guidelines on minimum standards of reporting? YesChoose an item.

Statistics

Are you able to assess all statistics in the manuscript, including the appropriateness of statistical tests used? Yes, and I have assessed the statistics in my report.

Quality of Written English

Please indicate the quality of language in the manuscript: Needs some language corrections before being published

Declaration of Competing Interests

Please complete a declaration of competing interests, considering the following questions:

- Have you in the past five years received reimbursements, fees, funding, or salary from an organisation that may in any way gain or lose financially from the publication of this manuscript, either now or in the future?
- Do you hold any stocks or shares in an organisation that may in any way gain or lose financially from the publication of this manuscript, either now or in the future?
- Do you hold or are you currently applying for any patents relating to the content of the manuscript?
- Have you received reimbursements, fees, funding, or salary from an organization that holds or has applied for patents relating to the content of the manuscript?
- Do you have any other financial competing interests?
- Do you have any non-financial competing interests in relation to this paper?

If you can answer no to all of the above, write 'I declare that I have no competing interests' below. If your reply is yes to any, please give details below.

I declare that I have no competing interests

I agree to the open peer review policy of the journal. I understand that my name will be included on my report to the authors and, if the manuscript is accepted for publication, my named report including any attachments I upload will be posted on the website along with the authors' responses. I agree for my report to be made available under an Open Access Creative Commons CC-BY license (http://creativecommons.org/licenses/by/4.0/). I understand that any comments which I do not wish to be included in my named report can be included as confidential comments to the editors, which will not be published.

I agree to the open peer review policy of the journal

To further support our reviewers, we have joined with Publons, where you can gain additional credit to further highlight your hard work (see: https://publons.com/journal/530/gigascience). On publication of this paper, your review will be automatically added to Publons, you can then choose whether or not to claim your Publons credit. I understand this statement.

Yes
